# A systematic review and meta-analysis of cohort studies on the potential association between NAFLD/MAFLD and risk of incident atrial fibrillation

**DOI:** 10.3389/fendo.2023.1160532

**Published:** 2023-07-05

**Authors:** Ben‐Gang Zhou, Sheng-Yong Ju, Yu-Zhou Mei, Xin Jiang, Meng Wang, Ai-Jing Zheng, Yan-Bing Ding

**Affiliations:** ^1^ Dalian Medical University, Dalian, Liaoning, China; ^2^ Department of Gastroenterology, Affiliated Hospital of Yangzhou University, Yangzhou, Jiangsu, China; ^3^ Medical Department, Affiliated Hospital of Yangzhou University, Yangzhou, Jiangsu, China; ^4^ Department of Gastroenterology, The People’s Hospital of China Three Gorges University, Yichang, Hubei, China; ^5^ Department of Neurology, The Third Clinical Medical College of China, Three Gorges University, Gezhouba Central Hospital of Sinopharm, Yichang, Hubei, China

**Keywords:** metabolic dysfunction-associated fatty liver disease, atrial fibrillation, systematic review, meta-analysis, cohort studies, non-alcoholic fatty liver disease

## Abstract

**Background and objective:**

The association between atrial fibrillation (AF) and non-alcoholic fatty liver disease (NAFLD) or metabolic-associated fatty liver disease (MAFLD) has been explored in recent cohort studies, however, the results have been controversial and inconclusive. This meta-analysis aimed to explore this potential association.

**Methods:**

We systematically searched PubMed, Embase, and Web of Science databases to identify all relevant cohort studies investigating the association between NAFLD/MAFLD and AF published from database inception to October 30, 2022. Random-effects models were utilized to calculate hazard ratios (HRs) with 95% confidence intervals (CIs) for summary purposes. Additionally, subgroup and sensitivity analyses were performed.

**Results:**

A total of 13 cohort studies with 14 272 735 participants were included. Among these, 12 cohort studies with 14 213 289 participants (median follow-up of 7.8 years) showed a significant association between NAFLD and an increased risk of incident AF (HR = 1.18, 95% CI: 1.12-1.23, *P* < 0.00001). Our subgroup analyses mostly yielded similar results, and the results of sensitivity analyses remained unchanged. However, meta-analysis of data from 2 cohort studies with 59 896 participants (median follow-up of 2.15 years) showed that MAFLD was not linked to incident AF (HR = 1.36, 95% CI: 0.63-2.92, *P* = 0.44).

**Conclusion:**

Current evidence shows that NAFLD may be linked to a slightly higher risk of developing AF, particularly among Asian populations and those diagnosed with NAFLD using FLI criteria. Nevertheless, there is not enough evidence to support the proposed association between MAFLD and an increased risk of AF. To better understand this relationship, future studies should consider factors such as specific population, the severity of NAFLD/MAFLD, diagnostic methods of NAFLD and AF, and cardiometabolic risk factors.

**Systematic Review Registration:**

https://www.crd.york.ac.uk/prospero, identifier CRD42022371503.

## 1 Introduction

With growing incidence of obesity and metabolic syndrome in recent years, non-alcoholic fatty liver disease (NAFLD) has emerged as the most prevalent liver disorder across the globe (1). Recent evidence reveals that the global prevalence of NAFLD in adults is estimated at 30%. Latin America reports the highest prevalence rate at 44%, followed by the Middle East and North Africa at 37%, South Asia at 34%, South-East Asia at 33%, North America at 31%, East Asia at 30%, Asia-Pacific at 28%, and Western Europe at 25% (2). NAFLD is a widespread condition that is identified by the presence of excessive fat accumulation in the liver of individuals without heavy alcohol consumption or other liver illnesses (3). NAFLD encompasses a variety of conditions, ranging from nonalcoholic fatty liver (NAFL) to the more severe nonalcoholic steatohepatitis (NASH), which can lead to liver cirrhosis and hepatocellular carcinoma (HCC) (4, 5). In the USA, UK, and France, NAFLD is already the most rapidly increasing cause of HCC (4). There is an increasing amount of evidence suggesting that NAFLD is multisystem disease, and its clinical and economic burden is not only confined to progressive liver disease (NASH, liver fibrosis, cirrhosis and HCC) but also linked to an increased risk of many extrahepatic diseases, such as cardiovascular disease (CVD), chronic kidney disease (CKD), diabetes mellitus (DM), and extrahepatic cancers (6–10).

Atrial fibrillation (AF) is the most frequently occurring persistent cardiac rhythm disorder and a prevailing reason for ischaemic stroke. AF is associated with an increased risk of mortality and morbidity stemming from stroke, congestive heart failure (CHF) and thromboembolism, and a decrease in overall quality of life, leading to a considerable healthcare cost and public health burden (11, 12). Common cardiovascular risk factors, such as smoking, obesity, hypertension, and DM, have been identified as the cause of 56.5% of new-onset AF cases (11). Considering that NAFLD and AF share common risk factors, the correlation between NAFLD and AF has become a subject of significant interest.

Based on our current knowledge, there have been seven meta-analyses (13–19) conducted previously to investigate the relationship between NAFLD and risk of AF. Nevertheless, five (13–17) of previous meta-analyses included cross-sectional and case-control design, and two meta-analyses (18, 19) of cohort studies involved a limited number of studies (up to seven cohort studies at most). In addition, the term metabolic (dysfunction) associated fatty liver disease (MAFLD) was put forward as a potential substitute for NAFLD by an international consensus in 2020 (20). In view of the differences between the criteria for NAFLD and MAFLD, previous meta-analyses did not evaluate the correlation between MAFLD and the likelihood of developing AF due to limited data. In the last two years, numerous high-quality cohort studies on the topic have been published, yet the results remain controversial and inconclusive. These newly studies have sparked our enthusiasm for updating existing evidence. Therefore, an updated systematic review and meta-analysis of cohort studies was carried out to determine the exact nature and extent of the correlation between NAFLD and the likelihood of developing AF.

## 2 Materials and methods

### 2.1 Registration of protocol

The protocol of this systematic review has been previously registered on PROSPERO platform with registration number CRD42022371503. Our study was carried out in accordance with the Preferred Reporting Items for Systematic Reviews and Meta-Analysis (PRISMA) statement (21) and the Meta-analysis Of Observational Studies in Epidemiology (MOOSE) reporting guidelines (22), as per our commitment to these guidelines.

### 2.2 Search strategy

A comprehensive search of PubMed, Embase, and Web of Science databases was conducted to identify all relevant cohort studies investigating the relationship between NAFLD/MAFLD and the likelihood of developing AF published from database inception to October 30, 2022, without language restrictions. The key words were as follow: “non-alcoholic fatty liver disease”, “nonalcoholic fatty liver disease”, “fatty liver”, “non-alcoholic fatty liver”, “nonalcoholic fatty liver”, “non-alcoholic steatohepatitis”, “nonalcoholic steatohepatitis”, “Metabolic dysfunction-associated fatty liver disease”, “Metabolic associated fatty liver disease”, “NAFLD”, “NAFL”, “NASH”, “MAFLD”, “atrial fibrillation”, “AF”. The search strategy utilized for PubMed can be found in online **Supplementary Table S1**, which was then modified to suit the Embase and Web of Science databases. Furthermore, supplementary studies were sought after by examining the references of all pertinent primary studies and review articles to guarantee comprehensiveness.

### 2.3 Study selection criteria

This meta-analysis only included studies that met the following inclusion criteria: (1) Observational cohort studies investigating the relationship between NAFLD/MAFLD and the likelihood of developing AF. (2) NAFLD was diagnosed through various methods including ultrasonography (USG) or computed tomography (CT) imaging techniques, liver biopsy, fatty liver index (FLI), or International Classification of Diseases (ICD) codes. These diagnoses were made only after ruling out excessive alcohol consumption and other potential causes of liver disease. To diagnose MAFLD, a combination of evidence is required. This includes histological evidence through liver biopsy, imaging, or blood biomarkers that indicate the presence of hepatic steatosis. Furthermore, the diagnosis is confirmed if there is either overweight/obesity, type 2 diabetes mellitus (T2DM), or metabolic dysregulation present. (3) Studies that have reported hazard ratios (HRs) or odds ratios (ORs) with 95% confidence intervals (CIs) for the outcome of interest, or the study provided sufficient raw data to calculate them. (4) Adult individuals (aged ≥18 years), regardless of their sex or ethnicity, were included in the meta-analysis. In instances where multiple studies were conducted on the same cohort or populations with some overlap, the one that provided the most in-depth data was taken into account. The study set out exclusion criteria that comprised of: (1) cross-sectional or case-control studies; (2) comments, editorials, letters, animal studies, reviews and meta-analyses; (3) studies with insufficient data; and (4) duplicate publications. In accordance with the aforementioned criteria, two investigators independently selected all the studies that met the eligibility requirements. Any discrepancies were resolved by discussing with each other.

### 2.4 Data extraction and quality assessment

Two authors extracted the data and assessed the methodological quality from each selected study. They worked independently and any differences were resolved through consensus. For each eligible study, data was extracted on the surname of first author, year of publication, nation of origin, study design, study subjects, participants characteristics, diagnostic methods of NAFLD and AF, follow-up time, ORs and HRs with their 95%CI, and adjusted confounders. The evaluation of methodological quality was carried out using the Newcastle-Ottawa Scale (NOS) (23) for cohort studies, which employs a star-based system (with a maximum of 9 stars) to evaluate a study that contains three domains: participants selection (with a maximum of four stars), comparability (with a maximum of two stars), and identification of outcomes of interest (with a maximum of three stars). The quality of the studies was assessed and classified into three groups: high quality (rated 7 stars or above), moderate quality (rated between 4 and 6 stars), and low quality (rated below 4 stars) (24).

### 2.5 Statistical analysis

The Cochrane Collaboration’s Review Manager software (Version 5.3, Copenhagen, Denmark) was utilized to conduct meta-analyses. Utilizing a random-effects model and the generic inverse variance method of DerSimonian and Laird (25), the estimation of effect size was calculated. Given the relatively low outcome of interest, ORs were treated as approximations of HRs because of the low incidence rates (26). For each eligible study, the effect size was determined by the HRs with 95% CIs. In case of report of adjusted ORs/HRs, the one that was adjusted for the greatest number of confounding variables was selected. To evaluate statistical heterogeneity among the studies, we utilized the Cochran’s Q-test (with a significance level of *P* < 0.10) and the I² statistic. Insignificant heterogeneity is represented by a value of I^2^ between 0-25%. Low heterogeneity is represented by a value of I^2^ between 26-50%. Moderate heterogeneity is represented by a value of I^2^ between 51-75%. Finally, high heterogeneity is represented by a value of I^2^ between 76-100% (27). When possible, we conducted subgroup analyses to investigate how certain study and participant characteristics affect the findings and elucidate potential causes of heterogeneity. Sensitivity analysis was conducted by excluding each of the included studies at a time to evaluate the possible excessive influence of individual studies on overall pooled estimates. To assess the possibility of publication bias, STATA/SE software (Version 12.0, STATA Corporation, Texas, USA) was used to examine the funnel plots, Begg’s test (28), and Egger’s test (29). A statistically significant result was considered when *P* < 0.05. If there is publication bias, a trim-and-fill method (30) was utilized to examine the influence of publication bias on the overall results.

## 3 Results

### 3.1 Study selection

**Figure 1:**
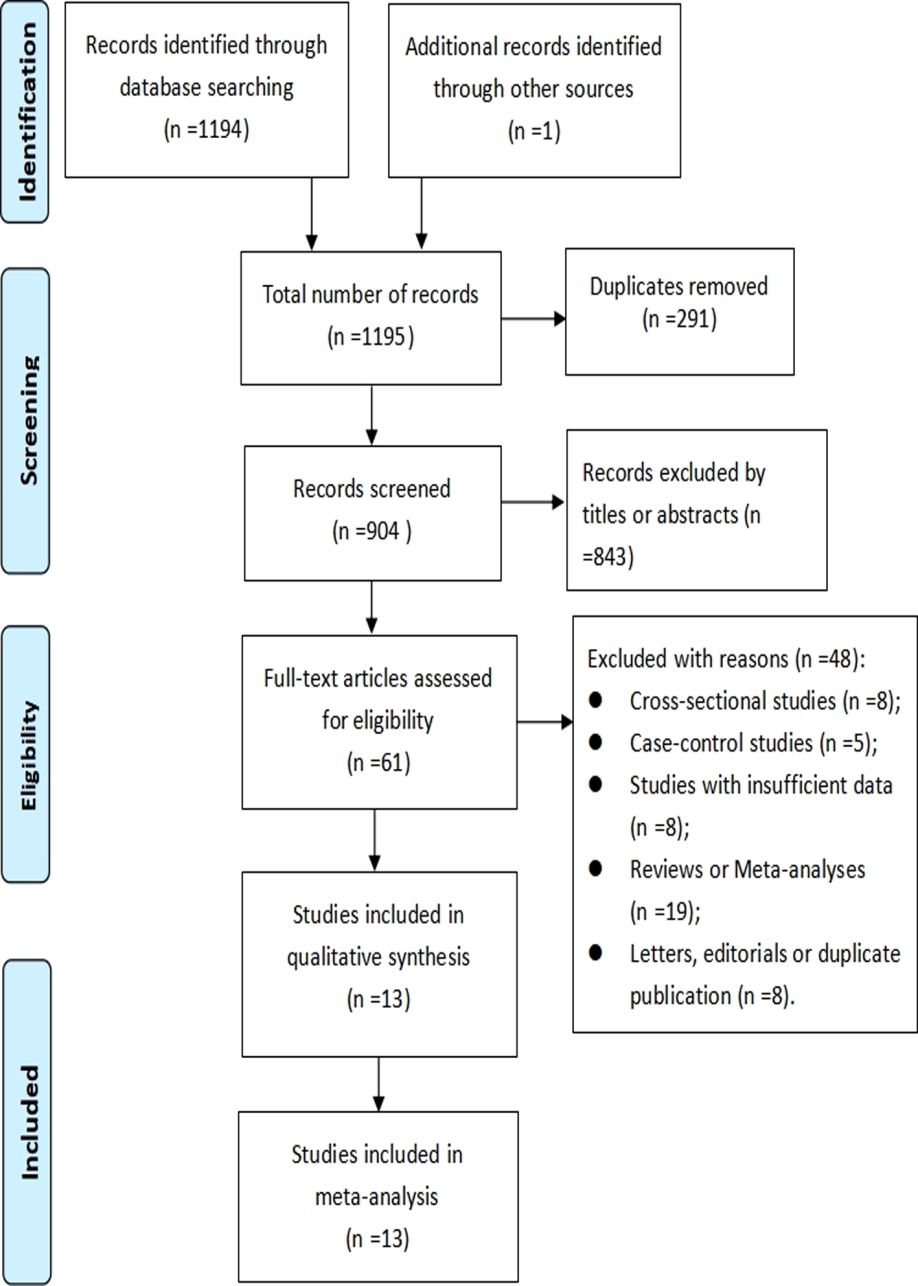
PRISMA flowchart of study selection process.

In total, 1195 records were found through the search. Upon removing duplicate publications and screening of titles and abstracts, 61 articles met the full-text screening criteria. Upon thorough examination of the remaining 61 articles that were potentially eligible, we proceeded to 48 articles with various reasons, which are described in detail in online **Supplementary Table S2**. Consequently, we identified 13 cohort studies (31–43) in our meta-analysis. The process of literature search and study selection is illustrated in **Figure 1**.

### 3.2 Study characteristics

**Table 1** provides a summary of the key attributes of the studies that were included. Thirteen longitudinal cohort studies with 14 272 735 adult participants were included in our meta-analysis. The range of publication period spanned from 2013 to 2022. One study (33) was presented in the form of an abstract, while the remaining studies were published in full-text. All the studies came from different countries across three continents. Six studies (31, 32, 36, 37, 40, 43) were conducted in Europe (Italy, Germany, UK, Netherlands), five (33, 38, 39, 41, 42) in Asia (Korea and China), two (34, 35) in North America (USA). The studies included sample sizes that varied from 400 to 8,048,055 participants, with a mean age range of 31 to 69 years old. Average follow-up time ranged from 2.1 to 16.3 years. Twelve studies (31–41, 43) explored the correlation between NAFLD and AF risk, while only two studies (42, 43) explored the correlation between MAFLD and AF risk. Regarding diagnostic methods of NAFLD, four studies (31, 32, 36, 43) used USG, one study (34) used CT, five (33, 38–41) used FLI, two (35, 37) used diagnostic codes (ICD codes). With respect to AF verification, most of studies were based on electrocardiogram (ECG), ICD codes and medical records. As shown in online **Supplementary Table S3**, the table summarizs the other characteristics (study continents, source of study subjects, adjusted confounders and corresponding data) of included studies. With regard to the methodological quality assessment, two studies were rated with five stars, indicating moderate quality, while nine studies received at least seven stars on the NOS, indicating high quality. The online **Supplementary Table S4** provides an in-depth assessment of the methodological quality of the studies included with the NOS.

**Table 1:** Main characteristics of included studies.

Study	Country	Participants	Sample size	Average age (years), male(%)	Diagnosis of NAFLD/MAFLD	AF verification	Follow-up time (mean years)	NOS score
Targher, 2013	Italy	Type 2 diabetic patients	400	64, 58.8	USG	ECG	10	8
Käräjämäki, 2015	Italy	Hypertensive patients with matched control	958	51, 47.0	USG	ECG	16.3	8
You, 2016	Korea	General population	232979	49, 36.3	FLI (≥30)	Diagnostic codes	3.7	5
Long, 2017	United States	General population	2060	59, 48.2	CT	ECG	12	8
Allen, 2019	United States	General population	19078	54, 47.6	ICD and HICDA codes	Medical records	7	9
Baratta, 2020	Italy	Patients with at least one comorbidity	898	56, 62.5	USG	Medical records	3.5	5
Labenz, 2020	Germany	General population	44096	55, 50.2	ICD codes	ICD codes	10	9
Roh, 2020	Korea	General population	334280	41, 48.3	FLI (>31)	ICD codes	5.3	8
Lee, 2021	Korea	General population	8048055	46, 52	FLI (≥60)	ICD codes	8.3	9
Zou, 2021	UK	General population	196128	56, 47.4	FLI (≥60)	ICD codes	8.0	8
Choi, 2022	Korea	General population	5333907	31, 57.0	FLI (≥60)	ICD codes	7.4	8
Lei, 2022	China	General population	54832	46, 63.2	Recent MAFLD criteria*	ECG	2.2	8
Van Kleef, 2022	Netherlands	General population	5064	69, 50.6	USG/recent MAFLD criteria*	ECG	2.1	7

NAFLD, nonalcoholic fatty liver disease; MAFLD, Metabolic dysfunction-associated fatty liver disease; AF, atrial fibrillation; NOS, Newcastle-Ottawa Scale; USG, Ultrasonography; ECG, electrocardiogram; CT, Computed tomography; FLI, Fatty liver index; ICD, international classification of diseases; HICDA, Hospital International Classification of Diseases Adapted; UK, United Kingdom; *the recent MAFLD diagnostic criteria based on steatosis together with overweight/obesity, diabetes or the presence of 2 minor metabolic dysfunction criteria.

### 3.3 NAFLD and risk of incident AF

**Figure 2:**
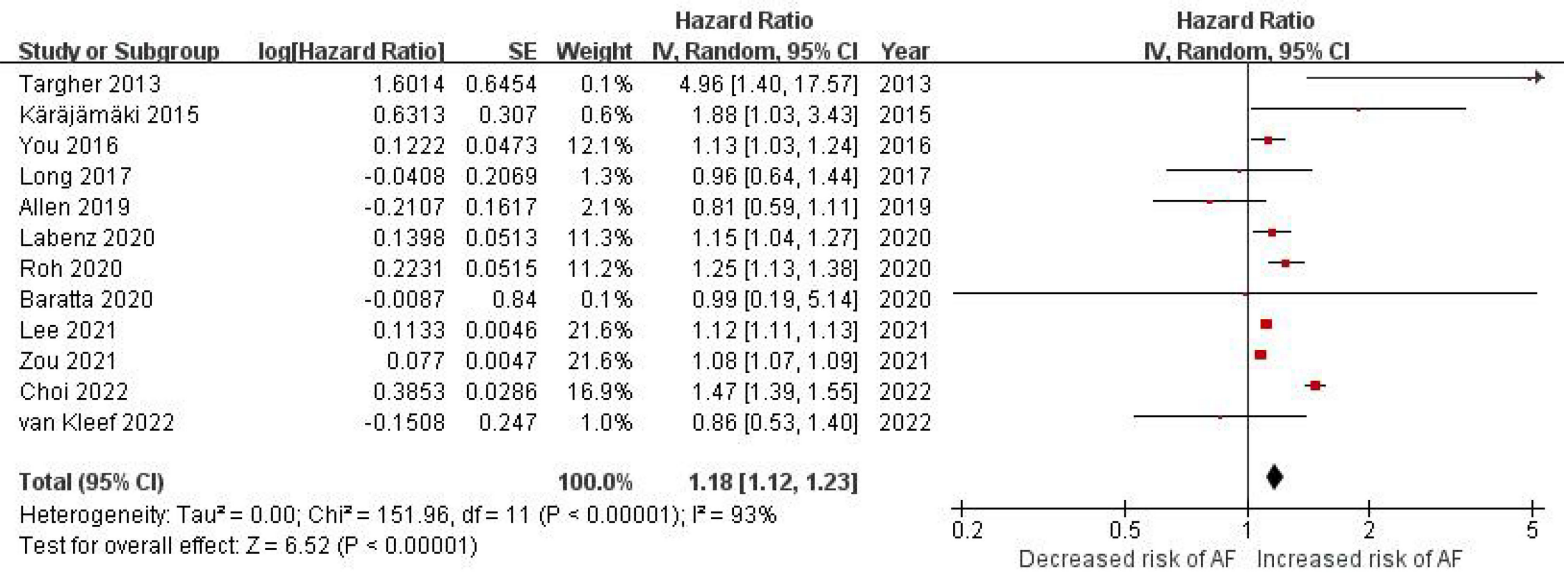
Forest plot of association between NAFLD and risk of incident AF.

As shown in **Figure 2**, twelve studies (31–41, 43) involving 14 213 289 participants evaluated the correlation between NAFLD and the risk of incident AF. Overall, the result of this meta-analysis showed that a significant correlation was observed between NAFLD and an increased incidence of AF (HR = 1.18, 95% CI: 1.12-1.23, *P* < 0.00001). Statistical heterogeneity was found to be high in this analysis (*I* ^2^ = 93%, *P* < 0.00001).

**Figure 3:**
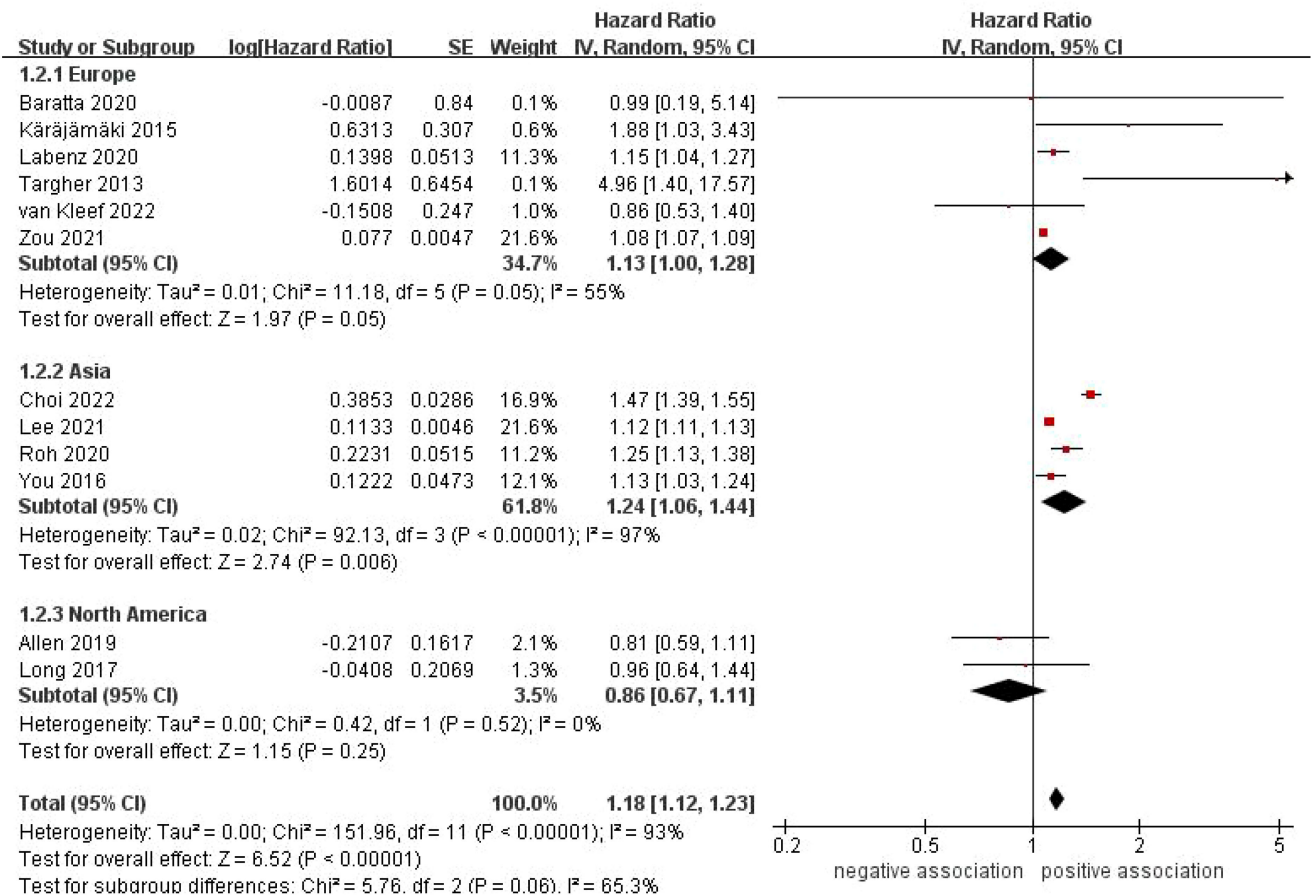
Forest plot of subgroup analysis based on study location.

**Figure 4:**
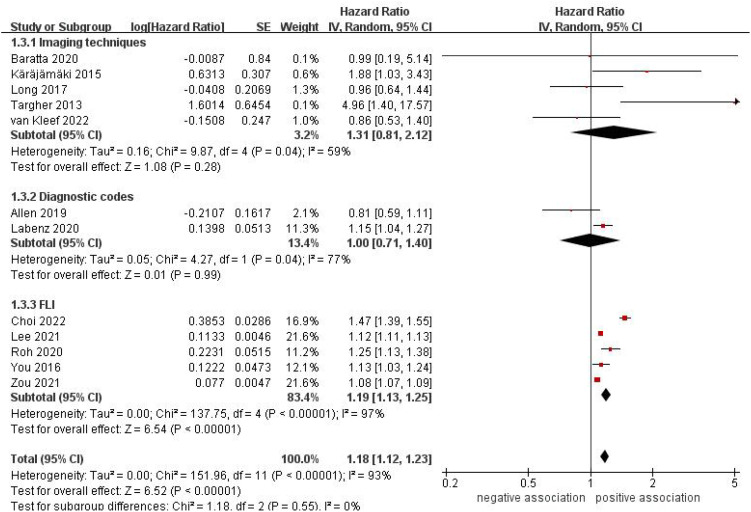
Forest plot of subgroup analysis based on diagnostic methods of NAFLD.

**Figure 5:**
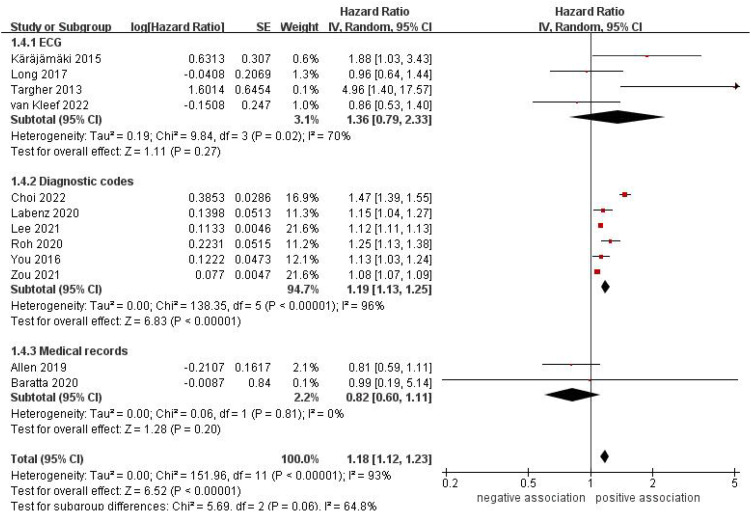
Forest plot of subgroup analysis based on methods of AF verification.

In order to investigate the potential reasons for heterogeneity among the studies included and possible factors affecting the overall results, we conducted numerous subgroup analyses in various ways. In a subgroup analysis based on study location, a significant correlation was identified between NAFLD and an increased incidence of AF in the subgroup from Asia (HR = 1.24, 95% CI: 1.06-1.44, *P* = 0.006; *I* ^2^ = 97%), whereas no significant association was observed between them in the subgroups from Europe (HR = 1.13, 95% CI: 1.00-1.28, *P* = 0.05; *I* ^2^ = 55%) and North America (HR = 0.86, 95% CI: 0.67-1.11, *P* = 0.25; *I* ^2^ = 0%) (**Figure 3**). In a subgroup analysis based on diagnostic methods of NAFLD, we found a significant correlation between NAFLD and the likelihood of developing AF when using FLI (HR = 1.19, 95% CI: 1.13-1.25, *P* <0.00001; *I* ^2^ = 97%) for diagnosing NAFLD. However, no significant association was observed when NAFLD was diagnosed using imaging techniques (HR = 1.31, 95% CI: 0.80-2.12, P = 0.28; *I* ^2^ = 59%) and diagnostic codes (HR = 1.00, 95% CI: 0.71-1.40, *P* = 0.99; *I* ^2^ = 77%) (**Figure 4**). In a subgroup analysis based on methods of AF verification, the results indicated a significant correlation between NAFLD and an increased risk of incident AF when using diagnostic codes (HR = 1.19, 95% CI: 1.13-1.25, *P* <0.00001; *I* ^2^ = 96%). However, no significant association was observed when ECG (HR = 1.36, 95% CI: 0.79-2.33, P = 0.27; *I* ^2^ = 70%) and medical records (HR = 0.82, 95% CI: 0.60-1.11, *P* = 0.20; *I* ^2^ = 0%) were utilized (**Figure 5**). In a subgroup analysis based on sample size, a significant connection between NAFLD and the incidence of AF was observed in studies that included more than 10,000 participants (HR = 1.18, 95% CI: 1.12-1.24, *P* <0.00001; *I* ^2^ = 96%), while studies with a sample size of less than 10000 did not reveal any significant association (HR = 1.31, 95% CI: 0.81-2.12, *P* = 0.28; *I* ^2^ = 59%) (online **Supplementary Figure S1**). In a subgroup analysis based on average age of participants, the risk of developing AF was found to be significantly associated with NAFLD in studies with an average age less than 55 years old (HR = 1.20, 95% CI: 1.04-1.39, *P* = 0.01; *I* ^2^ = 95%). However, the association was not significant in studies with an average age of 55 years old or older (HR = 1.10, 95% CI: 1.00-1.20, *P* = 0.05; *I* ^2^ = 39%) (online **Supplementary Figure S2**). In addition, we carried out subgroup analyses according to the follow-up time, study quality, number of gender, and adjustment for confounders. The associations between NAFLD and AF risk coincide with the overall pooled results in all these subgroups (online **Supplementary Figures S3–S6**). **Table 2** displays the results of subgroup analyses.

**Table 2:** Subgroup analyses of association between NAFLD and risk of incident AF.

Subgroup	No. of studies	HR (95%CI)	*P*_association_	*I^2^* (%)	*P*_heterogeneity_
Study location					
Europe	6	1.13 (1.00-1.28)	0.05	55	0.05
Asia	4	1.24 (1.06-1.44)	0.006	97	< 0.00001
North America	2	0.86 (0.67-1.11)	0.25	0	< 0.00001
Diagnostic methods of NAFLD					
Imaging techniques	5	1.31 (0.80-2.12)	0.28	59	0.04
Diagnostic codes	2	1.00 (0.71-1.40)	0.99	77	0.04
FLI	5	1.19 (1.13-1.25)	< 0.00001	97	< 0.00001
Methods of AF verification					
ECG	4	1.36 (0.79-2.33)	0.27	70	0.02
Diagnostic codes	6	1.19 (1.13-1.25)	< 0.00001	96	< 0.00001
Medical records	2	0.82 (0.60-1.11)	0.20	0	0.81
Follow-up time					
≥ 5 years	9	1.19 (1.13-1.25)	< 0.00001	95	< 0.00001
< 5 years	3	1.12 (1.02-1.23)	0.02	0	0.55
Sample size					
>10000	7	1.18 (1.12-1.24)	< 0.00001	96	< 0.00001
<10000	5	1.31 (0.81-2.12)	0.28	59	0.04
Average age of participants					
≥ 55 years	6	1.10 (1.00-1.20)	0.05	39	0.14
< 55 years	6	1.20 (1.04-1.39)	0.01	95	< 0.00001
Number of gender					
Male ≥ Female	6	1.22 (1.02-1.47)	0.03	95	< 0.00001
Male < Female	6	1.12 (1.02-1.23)	0.02	68	0.008
Study quality					
High-quality	10	1.18 (1.12-1.25)	< 0.00001	94	< 0.00001
Moderate-quality	2	1.13 (1.03-1.24)	0.01	0	0.88
Adjustment for confounders					
Adjusted	11	1.18 (1.12-1.23)	< 0.00001	93	< 0.00001
Unadjusted (Crude)	10	1.65 (1.34-2.04)	< 0.00001	98	< 0.00001

HR, hazard ratio; CI, confidence interval; NAFLD, nonalcoholic fatty liver disease; FLI, Fatty liver index; AF, atrial fibrillation; ECG, electrocardiogram.

To further validate our results, we conducted sensitivity analyses by excluding one study at a time. Our results demonstrated that the exclusion of any study did not have no significant effect on the overall risk of developing AF (**Table 3**). In addition, taking into account that the population of three studies (31, 32, 36) originated from special population with certain diseases, after excluding those three studies, we conducted a sensitivity analysis and found that our conclusions remained consistent, indicating that our findings were relatively robust.

**Table 3:** Results of sensitivity analyses.

Studies omitted	HR (95% CI)	*P*_association_	Heterogeneity
Targher, 2013	1.17 (1.12-1.23)	<0.00001	*I* ^2 ^= 93%, *P* < 0.00001
Käräjämäki, 2015	1.17 (1.12-1.23)	<0.00001	*I* ^2 ^= 93%, *P* < 0.00001
You, 2016	1.18 (1.12-1.25)	<0.00001	*I* ^2 ^= 93%, *P* < 0.00001
Long, 2017	1.18 (1.12-1.24)	<0.00001	*I* ^2 = ^= 93%, *P* < 0.00001
Allen, 2019	1.18 (1.13-1.24)	<0.00001	*I* ^2 ^= 93%, *P* < 0.00001
Baratta, 2020	1.18 (1.12-1.23)	<0.00001	*I* ^2 ^= 93%, *P* < 0.00001
Labenz, 2020	1.18 (1.12-1.24)	<0.00001	*I* ^2 ^= 93%, *P* < 0.00001
Roh, 2020	1.17 (1.11-1.23)	<0.00001	*I* ^2 ^= 93%, *P* < 0.00001
Lee, 2021	1.17 (1.03-1.32)	0.02	*I* ^2 ^= 93%, *P* < 0.00001
Zou, 2021	1.18 (1.05-1.32)	0.005	*I* ^2 ^= 91%, *P* < 0.00001
Choi, 2022	1.12 (1.08-1.16)	<0.00001	*I* ^2 ^= 80%, *P* < 0.00001
van Kleef, 2022	1.18 (1.12-1.24)	<0.00001	*I* ^2 ^= 93%, *P* < 0.00001

### 3.4 MAFLD and risk of incident AF

**Figure 6:**
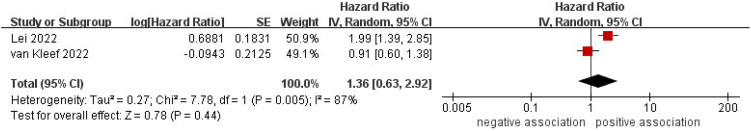
Forest plot of association between MAFLD and risk of incident AF.

Only two studies (42, 43) involving 59 896 participants reported data on the correlation between MAFLD and the incidence of AF. The study from Netherlands (43) with 5 064 individuals showed that MAFLD was not linked to incident AF (HR = 0.91, 95% CI: 0.60-1.38, *P* = 0.657). However, the study from China (42) with 54 832 participants showed that MAFLD may be linked to a higher risk of developing AF (HR = 1.99, 95% CI: 1.39-2.83, *P* < 0.001). The result of this meta-analysis indicated that MAFLD was not linked to incident AF (pooled HR = 1.36, 95% CI: 0.63-2.92, *P* = 0.44) with high heterogeneity (*I* ^2^ = 87%, *P* = 0.005) (**Figure 6**).

### 3.5 Evaluation for publication bias

Regarding the correlation between NAFLD and the likelihood of developing AF, the Begg’s funnel plot was slightly asymmetrical distribution (online **Supplementary Figure S7**). However, there is no indication of significant publication bias, as demonstrated by the Begg’s test and Egger’s test (*P_Begg_* = 0.732, *P_Egger_* = 0.302).

## 4 Discussion

### 4.1 Main findings and potential explanations

This updated meta-analysis (involving 13 cohort studies with 14.3 million adults) synthesized all current available evidence regarding the relationship between NAFLD/MAFLD and the likelihood of developing AF. Meta-analysis of data from 12 cohort studies with 14 213 289 participants (median follow-up of 7.8 years) showed that there was a significant association between NAFLD and a 1.18-fold increase in the risk of developing AF. Despite stratification by follow-up time, average age of participants, number of gender, study quality, or confounders adjustment, the magnitude of this risk remained substantially unchanged. The results of sensitivity analyses were robust. However, meta-analysis of data from 2 cohort studies with 59 896 participants (median follow-up of 2.15 years) showed that MAFLD was not linked to incident AF.

Considering that the prevalence of NAFLD varies in different locations, we further conducted subgroup analysis based on study location and found that a significant correlation was identified between NAFLD and an increased incidence of AF in Asia, but not in North American (n = 2 studies, HR = 0.86, 95% CI: 0.67-1.11) and Europe (n = 6 studies, HR = 1.13, 95% CI: 1.00-1.28, *P* = 0.05). This could be due to the limited sample size, resulting in insufficient statistical power. Thus, future studies with large samples should further confirm the relationship between NAFLD and AF in theses regions.

In this meta-analysis, the diagnostic methods of NAFLD based on imaging techniques (4 studies using USG, 1 using CT), FLI (5 studies), and diagnostic codes from databases (2 studies), the results of our subgroup analysis based on diagnostic methods of NAFLD indicated that NAFLD diagnosed by FLI had an elevated risk of developing AF, but NAFLD diagnosed by imaging techniques and diagnostic codes were not significantly associated with risk of incident AF. The findings suggest that the association between NAFLD identified by FLI and the risk of AF is considerably stronger. Currently, USG is the first-line imaging technique for diagnosing hepatic steatosis, with a high sensitivity and specificity for detecting moderate steatosis (44). Nevertheless, its sensitivity is not very accurate when the steatotic hepatocytes are less than 10%-12.5% (45, 46). Similarly, CT has been found to be more sensitive and specific for detecting moderate-to-severe liver steatosis. FLI, a surrogate measure for diagnosing NAFLD, provided good diagnostic accuracy (0.84) for fatty liver (47). Nevertheless, compared to liver biopsy, FLI is not as precise in detecting and assessing hepatic steatosis (44). Liver biopsy is commonly accepted as the gold standard for clinical diagnosis of NAFLD and could determine the severity of NAFLD, but it is not suitable for epidemiological studies (3). In order to gain a better understanding of the relationship between them, future studies should take advantage of more precise and practical diagnostic methods for NAFLD.

We also performed subgroup analyses based on methods of AF verification, sample size, and average age of participants. The result showed that a significant correlation between NAFLD and an increased risk of incident AF when using diagnostic codes for diagnosing AF, whereas no significant association was observed when using ECG and medical records. Similarly, there were no significant difference in studies with sample size less than 10 000 and average age of participants less than 50 years. The reason for this could be attributed to the limited number of studies and small sample size in these subgroups.

Given the difference between the diagnostic criteria of MAFLD and NAFLD, and the emphasis of MAFLD on the contribution of metabolic risk factors, our meta-analysis further analyzed the relationship MAFLD and the risk of incident AF, however, the result showed that no significant association was observed between them. In view of limited number studies and short follow-up time, future studies are need.

At present, the exact mechanisms of the relationship between NAFLD and an elevated risk of AF is yet to be elucidated. There are several potential explanations. First, systemic inflammation and oxidative stress may be significant factors in the development of AF in individuals with NAFLD. Low-grade systemic inflammation is a critical characteristic of NAFLD (7, 48). A proinflammatory milieu and oxidative stress can be caused by obesity, which is the result of a decreased release of the anti-inflammatory mediator adiponectin and an increased release of inflammatory mediators such as tumor necrosis factor (TNF)-α, interleukin (IL)-6, and C-reactive protein (CRP) from adipose tissue (49). Studies have demonstrated a link between heightened levels of proinflammatory biomarkers (plasma IL-6 and CRP) and a rise in incident AF (50, 51). Second, evidence of ectopic fat accumulation is frequently seen in other areas such as the myocardial, pericardial, epicardial and perivascular depots. These fat depots may act as an active endocrine and paracrine organ, secreting pro-inflammatory and vasoactive mediators which can lead to structural and functional damage to the myocardium (7, 52). Third, some studies indicated that NAFLD was linked to cardiac autonomic dysfunction, which may contribute to the incidence of AF (53–55). Fourth, adiponectin, an adipokine created mainly by adipocytes, is essential for maintaining metabolic and cardiovascular balance. Adiponectin may act as a bridge between adipose tissue, cardiac cells, and the vasculature, thereby playing a crucial role in this exchange (56, 57). NAFLD was linked to an increase in epicardial adipose tissue (EAT) (58). Being situated close to the myocardium and without fascia, EAT can directly affect the coronary arteries and myocardium through the paracrine actions of locally secreted adipocytokines and other bioactive molecules, thus contributing to the emergence of AF (56). Fifth, the relationship between NAFLD and cardiac remodeling and arrhythmia may be attributed to shared factors such as metabolic dysregulation and systemic insulin resistance induced by NAFLD (58).

Notably, our meta-analysis indicated that NAFLD may be linked to a slightly higher risk of developing AF. Subgroup analysis, however, revealed that this correlation was only significant in the Asian population, whereas it was not significant in the European and North American populations. We speculate that there are two possible reasons. One possible explanation was that compared to Caucasians, Hispanics, and African Americans, Asian NAFLD patients display more intense liver histological damage. Even though Asian people, such as those from South Korea, the Philippines, China, and India, had a lower BMI, their liver lobular inflammation and ballooning were more severe. Moreover, Asians living in the United States are more likely to suffer from severe liver steatosis and steatohepatitis than Caucasians (59). That is to say, Asian populations were more likely to experience liver tissue inflammation, which is a contributing factor to the development of AF. Another possible explanation was that the relatively small sample size in North America and Europe (but relatively large in Asia) could impact disparities in the likelihood of developing AF.

Current evidence suggests that the NAFLD spectrum is an emerging condition that may be associated with cardiovascular disease, including AF. Therefore, once their causal relationship is finally determined, a large number of patients with advanced liver disease will be candidates for anticoagulation, which is challenging in cirrhotic patients who exhibit an unstable hemostatic balance fluctuating between thrombosis and bleeding (60).

### 4.2 Comparison with previous studies

To our best knowledge, the present study is the most updated, largest and most comprehensive meta-analysis examining the relationship between NAFLD/MAFLD and the likelihood of developing AF to date. In 2017, Minhas et al. (13) conducted a meta-analysis of 3 observational studies (1 cross-sectional and 2 cohort) and showed that individuals with NAFLD have an increased likelihood of developing AF (OR = 2.47, 95% CI: 1.30-4.66, *P* = 0.005). In the same year, other two meta-analyses performed by Zhou et al. (14) (including 2 cross-sectional and 2 cohort studies) and Wijarnpreecha et al. (15) (including 2 cross-sectional and 3 cohort studies) yielded similar results. However, the above three meta-analyses (13–15) should be explained cautiously, since the data of cross-sectional studies and cohort studies were combined at the same time, and the sources of heterogeneity are not further comprehensively explored because of the limited number of studies included. In 2019, Mantovani et al. (16) performed a meta-analysis of 5 cross-sectional and 4 cohort studies, and the results indicated that NAFLD was linked to a higher likelihood of prevalent AF (OR = 2.07, 95% CI: 1.38-3.10) in cross-sectional studies, whereas the results of data from 4 cohort studies indicated that the occurrence of AF was not found to be significantly higher in individuals with NAFLD (HR = 1.34, 95% CI: 0.92-1.95). Further stratified analysis based on the type of cohort population found that NAFLD was only linked to a greater risk of incident AF in individuals with type 2 diabetes (n = 1 study; HR = 4.96, 95% CI:1.42-17.28). Whereafter, Gong et al. (17) conducted a larger meta-analysis including 1 case-control, 7 cross-sectional and 6 cohort studies, and the results indicated that NAFLD was independently linked to higher risks of AF (case-control and cross-sectional studies: OR = 1.71, 95% CI, 1.14-2.57; cohort studies: HR = 1.12, 95% CI: 1.11-1.13). Considering that different study designs may affect the relationship between NAFLD and AF, recent two meta-analyses (18, 19) only explored the relationship between them in cohort studies. In 2020, Cai and colleagues (18), who included 6 cohort studies involving 614 673 individuals, indicated that the presence of NAFLD was linked to a higher likelihood of developing incident AF after multivariable adjustment (RR= 1.19, 95% CI:1.07-1.31). Recently, Alon et al. (19) conducted a meta-analysis of 7 cohort studies involving 8 115 545 individuals and showed that the presence of NAFLD was linked to a higher likelihood of developing incident AF (OR = 1.27, 95%CI: 1.18-1.37). However, the results of this meta-analysis were mainly based on data from unadjusted confounding factors. Furthermore, all cohort studies included in the two meta-analyses mentioned above were included in our study.

Compared to seven aforementioned smaller meta-analyses, our updated meta-analysis confirm and further extend their findings. First, our updated meta-analysis included the most comprehensive cohort studies (n = 13 studies) and increases the total sample size (about 14.3 million), especially including newly published cohort studies that were not included in the previous meta-analysis (6 additional studies), thus providing the most up-to-date and sufficient epidemiologic evidence on the association between NAFLD/MAFLD and risk of incident AF. Second, we excluded cross-sectional and case-control study design because they are more susceptible to bias. Third, we further evaluated the association between MAFLD (a novel terminology) and risk of incident AF. Despite the limited amount of research conducted, definitive conclusions has yet to be determined.

### 4.3 Limitations

This present study is not without its limitations. Firstly, although most of the eligible studies were high-quality cohort studies, the causal relationship cannot be established because of the observational study design. Secondly, most of included studies adjusted for age, sex, obesity/body mass index, diabetes or fasting glucose, hypertension or blood pressure, and other cardiometabolic risk factors, but these adjusted factors were not consistent and incomplete adjustments among these studies. Furthermore, unmeasured or residual confounding factors cannot be ruled out. Thirdly, previous studies have suggested that liver stiffness may be related to the occurrence of AF (43), and liver fibrosis indexes were independently linked to risk of incident cardiovascular events (36). We speculate that evolutive NAFLD (liver fibrosis) may be more associated with AF, however, our study could not further analyze the severity of NAFLD (NASH, liver fibrosis or cirrhosis) and risk of incident AF due to a limited number of relevant data. Future research should address this issue. Fourthly, high statistical heterogeneity was observed in our meta-analysis, which limit the reliability of the conclusions of this study. We used random effects model to merge data because it captured uncertainty resulting from heterogeneity among studies (61). We also conducted numerous subgroup analyses and sensitivity analyses to explore the potential sources of statistical heterogeneity among the included studies. Different demographic characteristics of study populations, diagnostic methods of NAFLD and AF, severity of NAFLD, follow-up time, and adjustment for confounders may lead to this heterogeneity. Fifthly, the methods of AF verification were mainly based on standard ECG and ICD codes. Standard ECG may not accurately diagnose paroxysmal AF, which requires 24-hour Holter monitoring to diagnose. Misclassification bias may result from incorrect or insufficient coding of diagnostic codes (ICD codes) (37). This may have some impact on the results. Finally, the research evidence on the correlation between MAFLD and AF remains scarce, and the follow-up time was relatively short (approximately 2 years), since the term NAFLD has changed to MAFLD, future research should further explore the potential association in longer follow-up time.

## 5 Conclusions

Current updated evidence shows that NAFLD may be linked to a slightly higher risk of developing AF, particularly among Asian populations and those diagnosed with NAFLD using FLI criteria. Nevertheless, there is not enough evidence to support the proposed association between MAFLD and an increased risk of AF. To better understand this relationship, future studies should consider factors such as specific population, the severity of NAFLD/MAFLD, diagnostic methods of NAFLD and AF, and cardiometabolic risk factors.

## Data availability statement

The original contributions presented in the study are included in the article/**Supplementary Material**. Further inquiries can be directed to the corresponding author.

## Author contributions

B-GZ, S-YJ, Y-ZM, and Y-BD participated in the design of this study. B-GZ, Y-ZM, XJ collected and analyzed the data. MW and A-JZ sorted the data. B-GZ drafted the manuscript. Y-BD revised the manuscript. All authors have read and approved the final manuscript.
