# Supplementary material for: A systematic review and meta-analysis of cohort studies on the potential association between NAFLD/MAFLD and risk of incident atrial fibrillation
Source: Front Endocrinol (Lausanne). 2023 Jul 5;14:1160532. doi: 10.3389/fendo.2023.1160532 (PMC10355839; doi:10.3389/fendo.2023.1160532)
Supplement: Supplementary file 1 [file DataSheet_1.pdf]

Association between NAFLD/MAFLD and risk of incident atrial fibrillation: a systematic review and meta-analysis of cohort studies

Ben-Gang Zhou[1, 2], Sheng-Yong Ju[3], Yu-Zhou Mei[4], Xin Jiang[2], Meng Wang[5], Ai-Jing Zheng[4], Yan-Bing Ding[2*]

* Correspondence: Corresponding Author: Email: ybding@yzu.edu.cn.

Supplementary online material		
**Content**		**Page**
**Table S1.**	Representatives search strings for PubMed	2
**Table S2.**	Description of excluded studies	2-7
**Table S3.**	The other Characteristics of included studies	8-10
**Table S4.**	Methodological quality assessment of included studies with NOS	11
**Figure S1**.	Forest plot of subgroup analysis based on sample size	12
**Figure S2**.	Forest plot of subgroup analysis based on average age of participants	12
**Figure S3**.	Forest plot of subgroup analysis based on follow-up time	13
**Figure S4**.	Forest plot of subgroup analysis based on number of gender	13
**Figure S5**.	Forest plot of subgroup analysis based on study quality	14
**Figure S6**.	Forest plot of subgroup analysis based on adjustment for confounders	14
**Figure S7**.	Begg's funnel plot for association between NAFLD and AF	15

Table S1. Representatives search strings for PubMed (from inception to October 30, 2022)	
**Databases**	**Search strings**
**PubMed**	("non-alcoholic fatty liver disease"[MeSH Terms] OR "non-alcoholic fatty liver disease"[All Fields] OR "nonalcoholic fatty liver disease"[All Fields] OR "non-alcoholic fatty liver"[All Fields] OR "nonalcoholic fatty liver"[All Fields] OR "nonalcoholic steatohepatitis"[All Fields] OR "non-alcoholic steatohepatitis"[All Fields] "fatty liver"[MeSH Terms] OR "fatty liver"[All Fields] "Metabolic dysfunction-associated fatty liver disease"[All Fields] OR "Metabolic associated fatty liver disease"[All Fields] OR NAFLD[All Fields] OR NASH[All Fields] OR NAFL[All Fields] OR MAFLD[All Fields]) AND ("atrial fibrillation"[All Fields] OR "AF"[All Fields])

Table S2. Description of excluded studies at the stage of eligibility according to the PRISMA flow chart			
**No.**	**First author**	**Publication year**	**Reason for exclusion**
1.	Dauriz	2013	cross-sectional study
2.	Targher	2013	cross-sectional study
3.	Ding	2017	review
4.	Minhas	2017	meta-analysis
5.	Usman	2017	letter
6.	Wijarnpreecha	2017	meta-analysis
7.	Zhou	2017	meta-analysis
8.	Gholitabar	2018	studies with insufficient data
9.	Karajamaki	2018	review
10.	Mantovani	2018	letter
11.	Mantovani	2018	letter

12.	Zhang	2018	cross-sectional study
13.	Gong	2019	case-control
14.	Ismaiel	2019	meta-analysis
15.	Mahfouz	2019	case-control
16.	Mantovani	2019	meta-analysis
17.	Pastori	2019	studies with insufficient data
18.	Targher	2019	review
19.	Whitsett	2019	cross-sectional study
20.	Abd-Elsala	2020	cross-sectional study
21.	Abou Omar	2020	cross-sectional study
22.	Byeon	2020	case-control
23.	Cai	2020	letter
24.	Cai	2020	meta-analysis
25.	Haghbin	2020	review
26.	Kountouras	2020	letter
27.	Morningstar	2020	review
28.	Nersesov	2020	cross-sectional study
29.	Park	2020	cross-sectional study
30.	Pratama	2020	meta-analysis
31.	Targher	2020	review
32.	Zhang	2020	case-control
33.	Donnellan	2020	studies with insufficient data
34.	Pastori	2020	studies with insufficient data
35.	Bisaccia	2021	meta-analysis
36.	Gong	2021	meta-analysis
37.	Jiang	2021	studies with insufficient data
38.	Pastori	2021	case-control
39.	Polyzos	2021	review

40.	Van Kleef	2021	Duplicate publication
41.	Biccire	2022	review
42.	Chew	2022	review
43.	Chu	2022	studies with insufficient data
44.	Ma	2022	meta-analysis
45.	Tsai	2022	letter
46.	Chen	2022	editorial
47.	Decoin	2022	studies with insufficient data
48.	Wang	2022	studies with insufficient data

References for the table

1. Dauriz M, Mantovani A, Pichiri I, et al. Non-alcoholic fatty liver disease is associated with an increased prevalence of atrial fibrillation in patients with type 2 diabetes. Diabetes 2013, 62:A371.

2. Targher G, Mantovani A, Pichiri I, et al.Non-alcoholic fatty liver disease is associated with an increased prevalence of atrial fibrillation in hospitalized patients with type 2 diabetes. Clinical Science 2013, 125(6):301-309.

3. Ding YH,Ma Y,Qian LY,et al.Linking atrial fibrillation with non-alcoholic fatty liver disease:Potential common therapeutic targets.Oncotarget 2017,8(36):60673-60683.

4. Minhas AM,Usman MS,Khan MS,et al.Link Between Non-Alcoholic Fatty Liver Disease and Atrial Fibrillation:A Systematic Review and Meta-Analysis.Cureus 2017,9(4).

5. Usman MS,Siddiqi TJ.Emerging evidence for the association between non-alcoholic fatty liver disease and cardiac arrhythmias.Digestive and Liver Disease 2017,49(10):1166.

6. Wijarnpreecha K,Boonpheng B,Thongprayoon C,et al.The association between non-alcoholic fatty liver disease and atrial fibrillation: A meta-analysis. Clinics and Research in Hepatology and Gastroenterology 2017,41(5):525-532..

7. Zhou Y,Lai C,Peng C,et al.Nonalcoholic fatty liver disease as a predictor of atrial fibrillation:A systematic review and meta-analysis.Postepy w Kardiologii Interwencyjnej 2017,13(3):250-257.

8. Gholitabar F,Lee S,Bakir M,et al.Non-alcoholic fatty liver disease is associated with onset of atrial fibrillation at a younger age:A nationwide study.Journal of the American College of Cardiology 2018,71(11)

9. Karajamaki AJ,Hukkanen J,Ukkola O:The association of non-alcoholic fatty liver disease and atrial fibrillation: a review.Annals of Medicine 2018,50(5):371-380..

10. Mantovani A:NAFLD and risk of cardiac arrhythmias: Is hyperuricemia a neglected pathogenic mechanism? Digestive and Liver Disease 2018,50(5):518-520..

11. Mantovani A,Nascimbeni F:Is it time to include non-alcoholic fatty liver disease in the current risk scores for atrial fibrillation? Digestive and Liver Disease 2018,50(6):626-628.

12. Zhang Y,Li P,Miao M,et al.Nonalcoholic Fatty Liver Disease Is Associated with Increased Atrial Fibrillation Risk in an Elderly Chinese Population:A Cross-Sectional Study. Biomed Research International 2018,2018.

13. Gong F,Zhou J,Liu F,et al.Relationship between nonalcoholic fatty liver disease and arrhythmia on electrocardiogram. Journal of International Translational Medicine 2019,7(4):122-126.

14. Ismaiel A,Colosi HA,Rusu F,et al.Cardiac Arrhythmias and Electrocardiogram Modifications in Non-Alcoholic Fatty Liver Disease. A Systematic Review.J Gastrointestin Liver Dis 2019,28(4):483-493.

15. Mahfouz RA,Gouda M,Galal I,et al.Interatrial septal fat thickness and left atrial stiffness are mechanistic links between nonalcoholic fatty liver disease and incident atrial fibrillation. Echocardiography 2019, 36(2):249-256.

16. Mantovani A,Dauriz M,Sandri D,et al.Association between non-alcoholic fatty liver disease and risk of atrial fibrillation in adult individuals:An updated meta-analysis. Liver International 2019,39(4):758-769.

17. Pastori D,Sciacqua A,Marcucci R,et al.Non-alcoholic fatty liver disease and atrial fibrillation. Prevalence and impact on clinical outcomes.Circulation 2019,140.

18. Targher G.Risk of atrial fibrillation in patients with nonalcoholic steatohepatitis. Liver International 2019, 39(5):818-820.

19. Whitsett M,Wilcox J,Yang A,et al.Atrial fibrillation is highly prevalent yet undertreated in patients with biopsy-proven nonalcoholic steatohepatitis. Liver International 2019,39(5):933-940.

20. Abd-Elsalam S,Abou Omar MA,Yousef M.Nonalcoholic fatty liver disease (NAFLD) and the risk of atrial fibrillation. Hepatology International 2020,14:S354.

21. Abou Omar MA,Alaarag A,Abd-Elsalam S,et al.Nonalcoholic fatty liver disease and the risk of atrial fibrillation.Open Access Macedonian Journal of Medical Sciences 2020,8:530-535.

22. Byeon JR,Park J,Lee HA,et al,Nonalcoholic fatty liver disease in patients with atrial fibrillation: Clinical characteristics and link with cardiovascular risk. Hepatology 2020,72(1 SUPPL):983A.

23. Cai X,Zheng S,Liu Y,et al.Nonalcoholic fatty liver disease is associated with increased risk of atrial fibrillation. Liver International 2020,40(7):1594-1600.

24. Cai X,Zheng S,Zhang Y,et al.Helicobacter pylori, non-alcoholic liver disease and atrial fibrillation:Is there a link? Liver International 2020,40(8):2037-2038

25. Haghbin H,Gangwani MK,Ravi JK,et al.Nonalcoholic fatty liver disease and atrial fibrillation: possible pathophysiological links and therapeutic interventions.Annals of Gastroenterology 2020,33(6):603-614.

26. Kountouras J,Doulberis M,Papaefthymiou A,et al.Impact of Helicobacter pylori-linked metabolic syndrome on non-alcoholic fatty liver disease and its connected atrial fibrillation risk Liver International 2020, 40(8):2036-2037.

27. Morningstar JE,Syn WK,Litwin SE.The Emerging Epidemic of Nonalcoholic Fatty Liver Disease and

Cardiovascular Risk:True, True, and Related? Digestive Diseases and Sciences 2020,65(7):1885-1887.

28. Nersesov A,Rakisheva A,Mussagaliyeva A,et al.Characteristics of concurrent cardiovascular diseases in patients with NAFLD.Hepatology International 2020,14:S358.

29. Park HE,Lee H,Choi SY,et al.The risk of atrial fibrillation in patients with non-alcoholic fatty liver disease and a high hepatic fibrosis index.Scientific reports 2020,10(1):5023.

30. Pratama PB,Nugraha PF.Arrhythmia as extrahepatic complication in patients with nonalcoholic fatty liver disaese:A systematic review and metaanalysis.Hepatology International 2020,14:S345.

31. Targher G,Byrne CD,Tilg H.NAFLD and increased risk of cardiovascular disease: Clinical associations, pathophysiological mechanisms and pharmacological implications.Gut 2020,69(9):1691-1705..

32. Zhang X,Chen G-Y,Wang Z-X,et al.Nonalcoholic fatty liver disease impacts the control of the international normalized ratio in patients with atrial fibrillation. Annals of Translational Medicine 2020,8(16).

33. Donnellan E,Cotter TG,Wazni OM,et al.Impact of Nonalcoholic Fatty Liver Disease on Arrhythmia Recurrence Following Atrial Fibrillation Ablation. JACC:Clinical Electrophysiology 2020,6(10):1278-1287..

34. Pastori D,Sciacqua A,Marcucci R,et al.Prevalence and Impact of Nonalcoholic Fatty Liver Disease in Atrial Fibrillation. Mayo Clin Proc 2020,95(3):513-520.

35. Bisaccia G,Ricci F,Melchiorre E,et al.Cardiovascular morbidity and mortality related to non-alcoholic fatty liver disease: A systematic review and meta-analysis of prospective studies.European Heart Journal, Supplement 2021,23(SUPPL G):G103.

36. Gong H,Liu X,Cheng F.Relationship between non-alcoholic fatty liver disease and cardiac arrhythmia:a systematic review and meta-analysis. Journal of International Medical Research 2021,49(9).

37. Jiang Y,Chowdhury S,Xu B,et al.IS NONALCOHOLIC FATTY LIVER DISEASE ASSOCIATED WITH WORSE INPATIENT OUTCOMES IN PATIENTS WITH ATRIAL FIBRILLATION? A NATIONWIDE INPATIENT SAMPLE ANALYSIS.Gastroenterology 2021,160(6):S-834.

38. Pastori D,Sciacqua A,Marcucci R,et al.Non-alcoholic fatty liver disease (NAFLD),metabolic syndrome and cardiovascular events in atrial fibrillation.A prospective multicenter cohort study. Internal and emergency medicine 2021,16(8):2063-2068.

39. Polyzos SA,Kechagias S,Tsochatzis EA.Review article:non-alcoholic fatty liver disease and cardiovascular diseases:associations and treatment considerations. Alimentary Pharmacology and Therapeutics 2021, 54(8):1013-1025.

40. Van Kleef LA,Lu Z,Kavousi M,et al.Liver stiffness,but not non-alcoholic fatty liver disease, is associated with atrial fibrillation:The rotterdam study.Hepatology 2021, 74(SUPPL 1):1022A-1023A.

41. Biccire FG,Bucci T,Menichelli D,et al.Mediterranean Diet:A Tool to Break the Relationship of Atrial Fibrillation with the Metabolic Syndrome and Non-Alcoholic Fatty Liver Disease.Nutrients 2022,14(6).

42. Chew NWS,Chong B,Ng CH,et al.The genetic interactions between non-alcoholic fatty liver disease and cardiovascular diseases.Frontiers in Genetics 2022,13..

43. Chu Y,Yu F,Wu Y,et al.Identification of genes and key pathways underlying the pathophysiological association between nonalcoholic fatty liver disease and atrial fibrillation. BMC Medical Genomics 2022,15(1).

44. Ma T,Yu X,Sun M.Relationship between non-alcoholic fatty liver disease and atrial fibrillation: assessment of latest evidence.Journal of hepatology 2022.

45. Tsai WC,Yu ML,Dai CY.Liver stiffness, fatty liver disease and atrial fibrillation in the Rotterdam study: Some issues. Journal of Hepatology 2022,77(5):1466-1467.

46. Chen J,Mei Z,Wang Y,et al.Causal effect of non-alcoholic fatty liver disease on atrial fibrillation. European Journal of Internal Medicine 2022,105:114-117.

47. Decoin R,Butruille L,Defrancq T,et al.High liver fibrosis scores in metabolic dysfunction-associated fatty liver disease patients are associated with adverse atrial remodeling and atrial fibrillation recurrence following catheter ablation. Frontiers in Endocrinology 2022,13.

48. Wang Z,Wang Y,Luo F et al.Impact of advanced liver fibrosis on atrial fibrillation recurrence after ablation in non-alcoholic fatty liver disease patients. Frontiers in cardiovascular medicine 2022, 9:960259-960259.

Table S3 The other characteristics of included studies

First author, year	Continents	Study subjects	Confounders adjustment	OR/HR(95%CI)
Targher, 2013	Europe	400 Italian type 2 diabetic patients without previous history of AF,heart valve disease and known causes of chronic liver diseases	Age, sex, BMI, SBP, hypertension treatment, history of heart failure, electrocardiographic PR interval and left ventricular hypertrophy	aOR:4.96(1.40-17.0)
Käräjämäki, 2015	Europe	985 middle-aged hypertensive subjects were randomly selected from the Finnish National Insurance Register (OPERA Study) without differences in age or sex and with no previous history of atrial fibrillation and no known cause of chronic liver disease	Age ,sex, diabetes, study group status (hypertensive vs. control), BMI, saist circumference, alcohol consumption, smoking, serum ALT concentration, SBP, quick index, LVMI (Left Ventricular Mass Index), left atrial diameter, ANP, CAD and hs-CRP.	aHR:1.88(1.03-3.45)
You, 2016	Asian	A total of 232,979 adult subjects with no prior history of atrial fibrillation,structural heart disease,or alcohol abuse were enrolled from the Korea National Health Insurance Service National Sample Cohort between 2009 and 2013	Age, sex, obesity, elevated , impaired fasting glucose, Dyslipidemia, heart failure, serum creatinine.	aHR:1.13(1.03-1.24)
Long, 2017	North America	2060 middle-aged Americans from the Framingham Heart Study with no history of atrial fibrillation and chronic liver disease	Sex, age, BMI, SBP, DBP, current smoking, use of antihypertensive medication, prevalent diabetes mellitus, history of heart failure, history of myocardial infarction	aHR:0.96(0.64-1.45)
Allen, 2019	North America	All adults diagnosed with NAFLD in Olmsted County, Minnesota,between 1997 and 2014 were included,and an age-and sex-matched (1:4) reference cohort was selected from the general population	Age, sex, BMI, time-dependent smoking, diabetes mellitus, hypertension, dyslipidemia	aHR:0.81(0.59-1.11)

Baratta, 2020	Europe	A total of 898 patients admitted to the Internal Medicine and Metabolic Disorders Day Service at the Policlinico Umberto I University Hospital had at least one of the following cardiac metabolic diseases: arterial hypertension, overweight/obesity, type 2 diabetes, dyslipidemia, AF or metabolic syndrome	Not reported	cOR:0.99(0.19-5.14)
Labenz, 2020	Europe	Patients diagnosed with NAFLD/NASH for the first time between January 2000 and December 2015 at 1262 general hospitals in Germany were enrolled	Age, sex, treating physician, type 2 diabetes, arterial hypertension, hyperlipidemia	aHR:1.15(1.04-1.26)
Roh, 2020	Asian	A total of 334,280 healthy individuals without complications who underwent national health examination in South Korea between 2009 and 2014 were included	Age, sex, clinical characteristics, diabetes, hypertension, Heart failure, myocardial infarction	aHR:1.55(1.19-2.03)
Lee, 2021	Asian	A total of 8,048,055 patients who underwent national health examinations in South Korea from 2009 to 2017 were included	Age, sex, hypertension, Diabetes, Dyslipidemia, CKD, Smoking, alcohol consumption, exercise, low income, SBP, Total cholesterol, fasting glucose.	aHR:1.12(1.11-1.13)
Zou, 2021	Europe	196,128 UK adults aged 40–69 were included in the UK Biobank Database between 2006 and 2010	Age, sex, race, assessment center, smoking, townsend index, SBP, DBP, Non-HDL cholesterol, Anti-hypertensive treatment, lipid-lowering treatment, HbA1c, Anti-diabetic treatment	aHR:1.08(1.07-1.09)
Choi, 2022	Asian	A total of 5,333,907 individuals aged 20-39 years who underwent health check-ups at the National Health Insurance Company of Korea between January 2009 and December 2012 were included	Age, sex, hypertension, diabetes mellitus, dyslipidemia, Heart failure, prior ischemic stroke, prior myocardial infarction, chronic obstructive pulmonary disease, CKD, Sleep apnea, hyperthyroidism, smoking, alcohol	aHR:1.47(1.39-1.55)

			consumption, low income.	
Lei, 2022	Asian	54832 pariticipants from 5 health management centers in Hubei province between January 2009 to December 2017	Age, sex, self-reported smoking, self-reported drinking, red blood cell, leukocyte count, haemoglobin, platelet count, CKD and medical center as random effect.	aHR:1.99(1.39-2.83)
Van Kleef, 2022	Europe	Participants that had visited the Rotterdam Study research center between March 2009 and June 2014 and had undergone abdominal ultrasound	Age, sex, alcohol consumption, smoking, education, high waist circumference, hypertension, hypo-HDL, Hypertriglyceridemia, (Pre)diabetes, coronary heart disease and heart failure	NAFLD: aHR: 0.86(0.53-1.38) MAFLD: aHR: 0.91(0.60-1.38)
Abbreviations: AF, atrial fibrillation; aHR, adjustedhazard ratio; ALT, alanine aminotransferase; ANP, atrial natriuretic peptide; aOR, adjusted odds ratio; BMI, body mass index; CAD, coronary artery disease; CKD, chronic kidney disease; cOR, crude odds ratio; DBP, diastolic blood pressure; HDL, high-density lipoprotein; hs-CRP, high-sensitive C-reactive protein; NAFLD, nonalcoholic fatty liver disease; NASH, nonalcoholic steatohepatitis; MAFLD, Metabolic dysfunction-associated fatty liver disease; SBP, systolic blood pressure; UK, United Kingdom.				

Table S4 Methodological quality assessment of included studies with NOS					
First author (publication year)	Selection	Comparability	Outcome	Total (0-9)	Quality
Targher (2013)	☆☆☆	☆☆	☆☆☆	8	High
Käräjämäki (2015)	☆☆☆	☆☆	☆☆☆	8	High
You (2016)	☆☆	☆☆	☆	5	Moderate
Long (2017)	☆☆☆	☆☆	☆☆☆	8	High
Allen (2019)	☆☆☆☆	☆☆	☆☆☆	9	High
Baratta (2020)	☆☆☆	–	☆☆	5	Moderate
Labenz (2020)	☆☆☆☆	☆☆	☆☆☆	9	High
Roh (2020)	☆☆☆	☆☆	☆☆☆	8	High
Lee (2021)	☆☆☆☆	☆☆	☆☆☆	9	High
Zou (2021)	☆☆☆	☆☆	☆☆☆	8	High
Choi (2022)	☆☆☆	☆☆	☆☆☆	8	High
Lei (2022)	☆☆☆☆	☆☆	☆☆	8	High
Van Kleef (2022)	☆☆☆	☆☆	☆☆	7	High
Note: NOS, Newcastle–Ottawa Scale; "- "stands for zero point.					

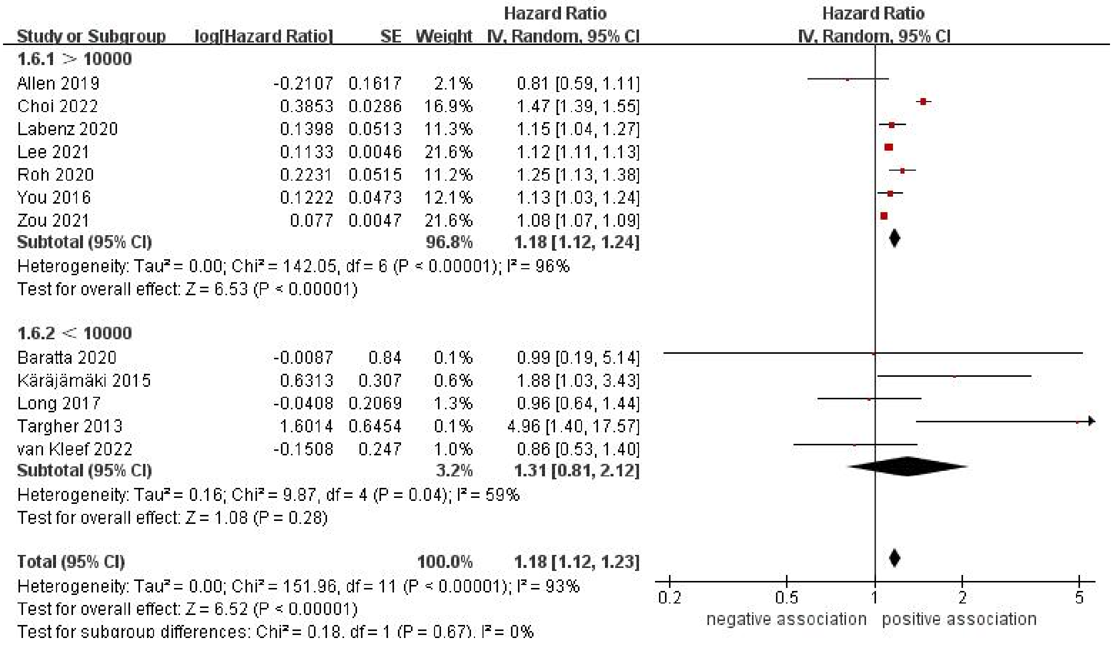

Figure S1. Forest plot of subgroup analysis based on sample size

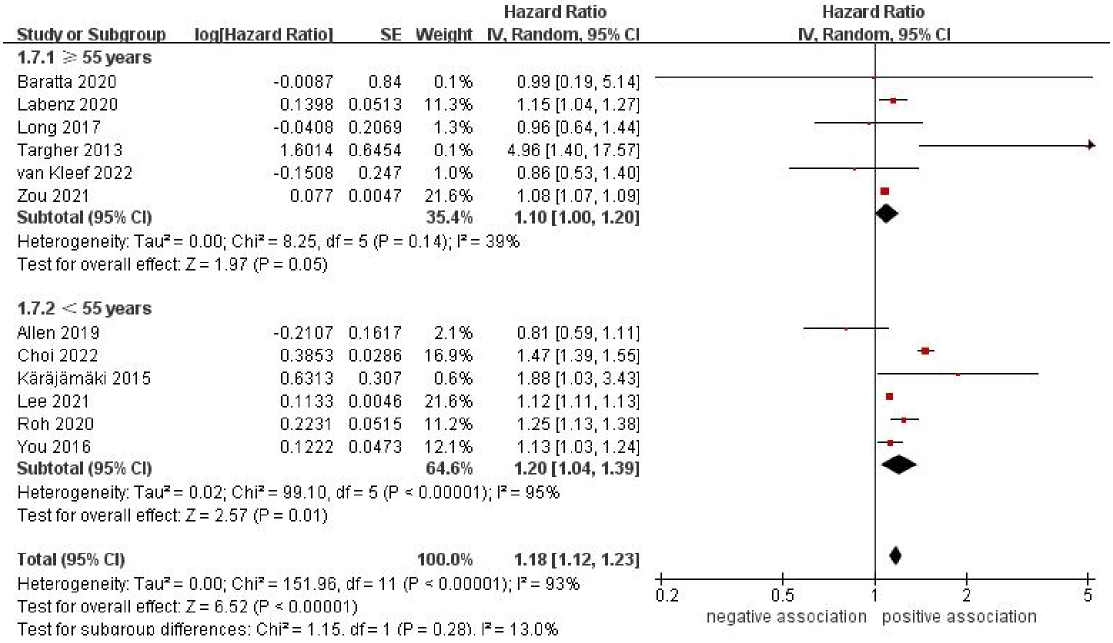

Figure S2. Forest plot of subgroup analysis based on average age of participants

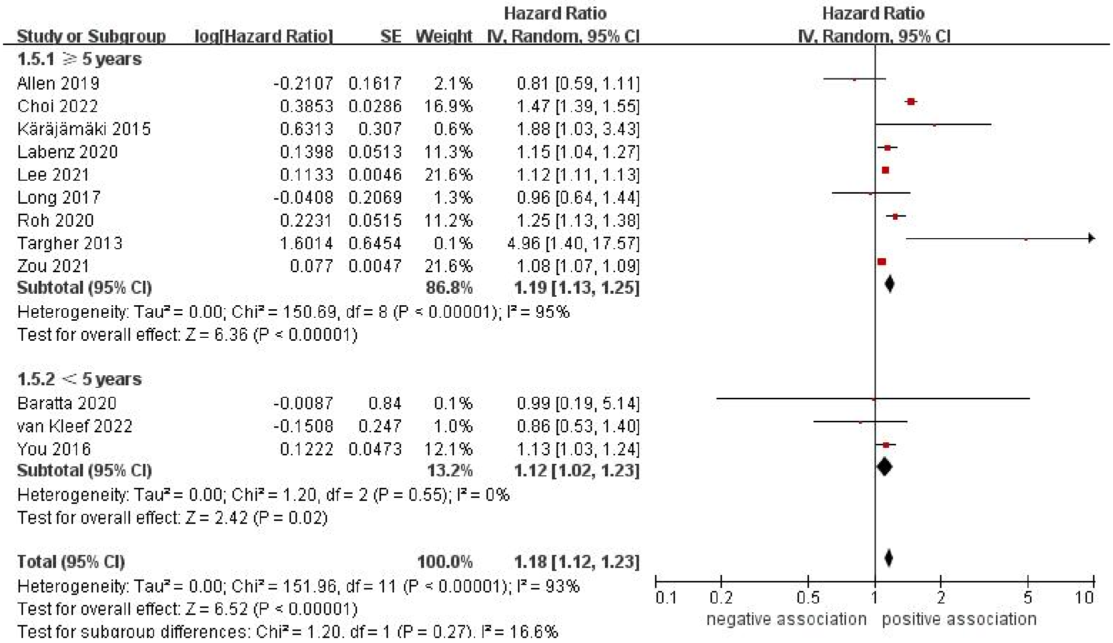

Figure S3. Forest plot of subgroup analysis based on follow-up time

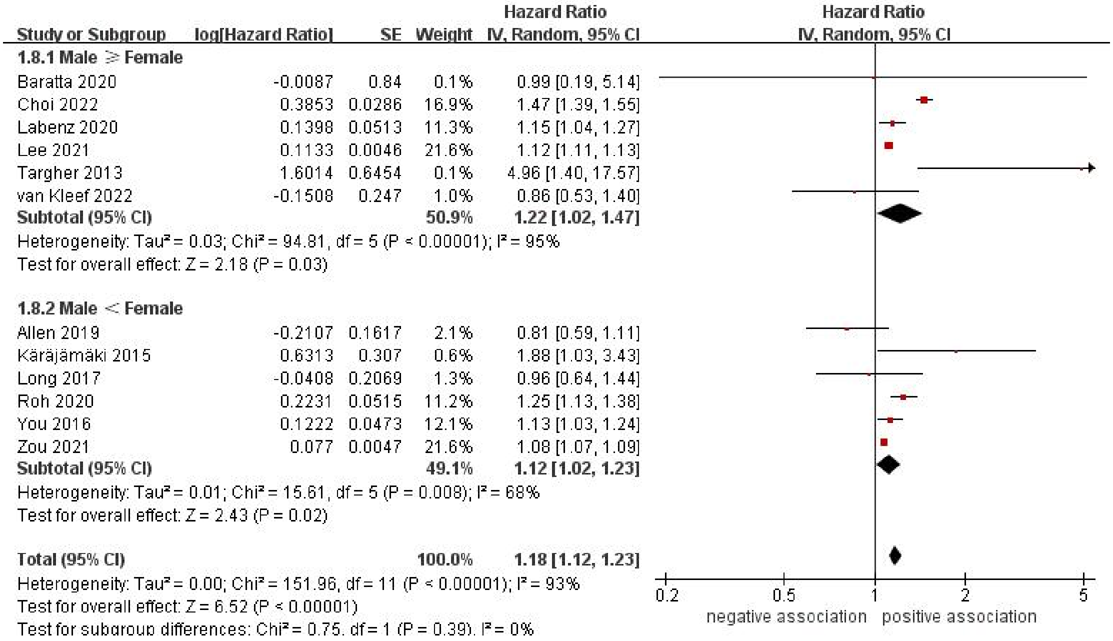

Figure S4. Forest plot of subgroup analysis based on number of gender

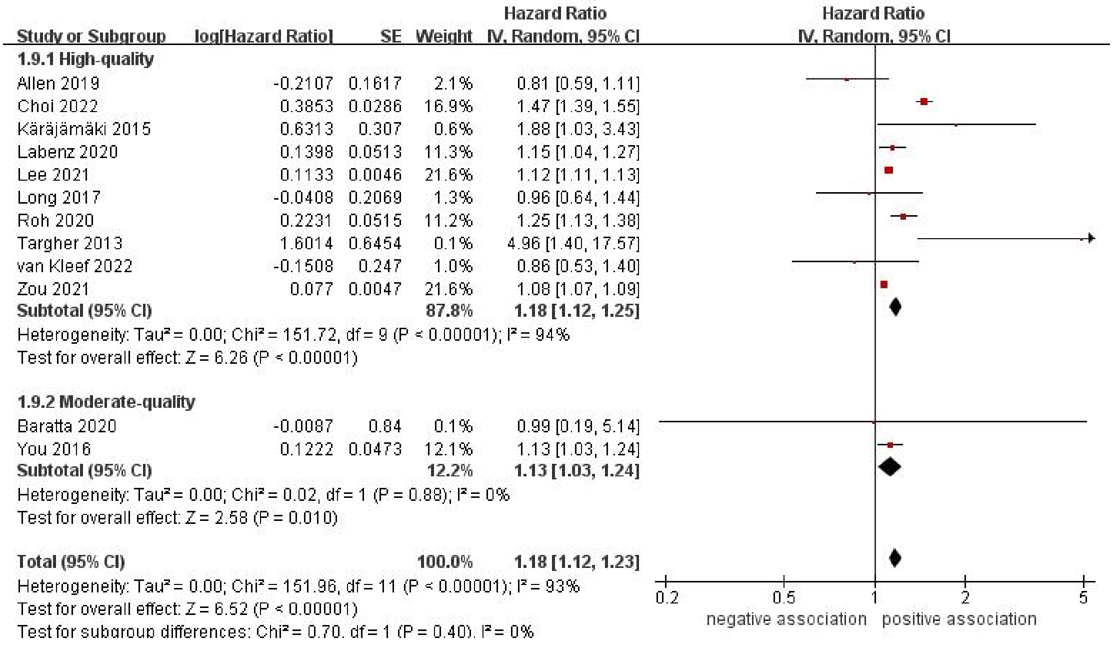

Figure S5. Forest plot of subgroup analysis based on study quality

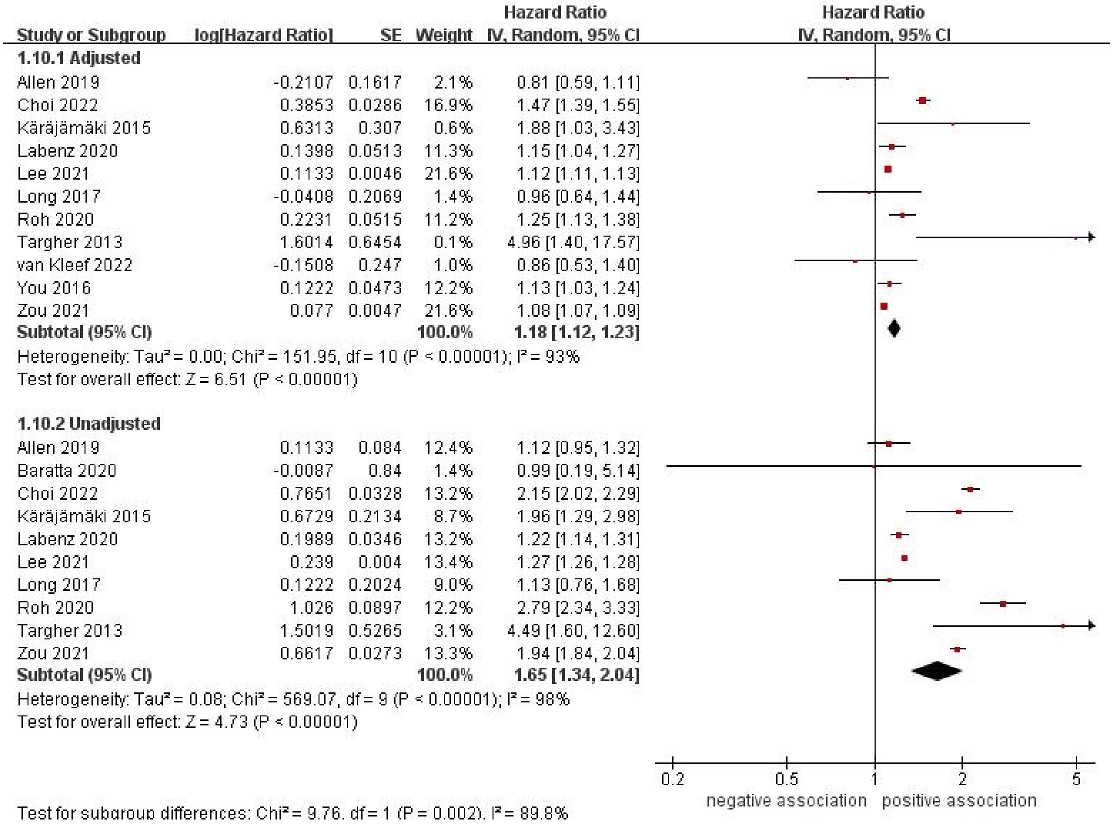

Figure S6. Forest plot of subgroup analysis based on adjustment for confounders

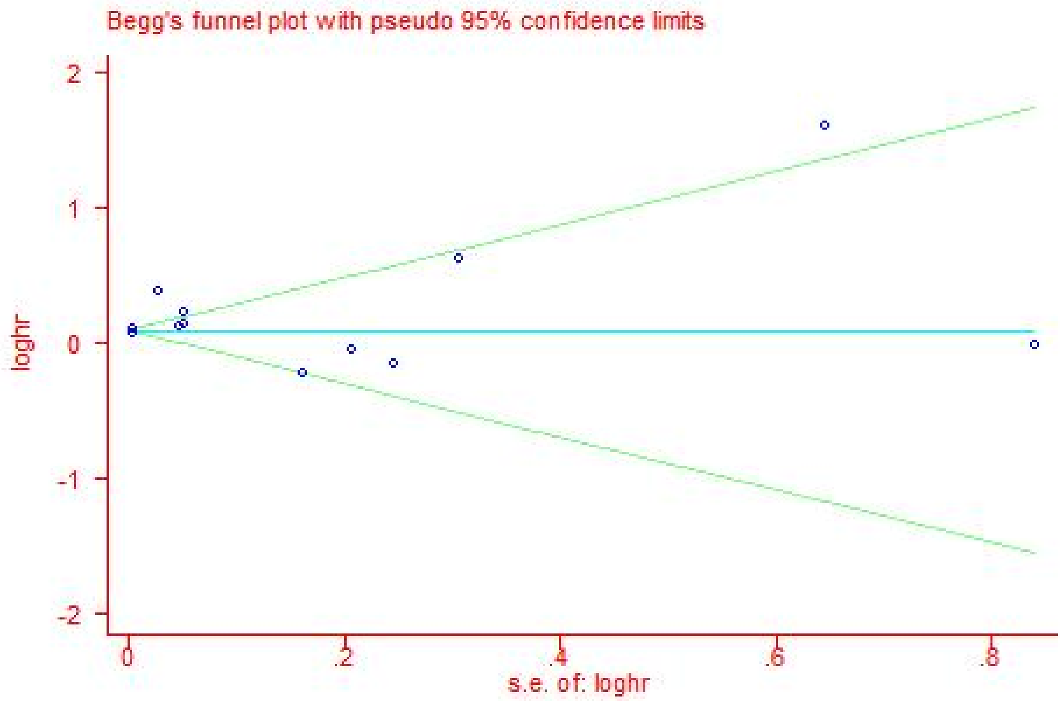

Figure S7. Begg's funnel plot for association between NAFLD and AF
